# High Daytime Temperature Responsive MicroRNA Profiles in Developing Grains of Rice Varieties with Contrasting Chalkiness

**DOI:** 10.3390/ijms241411631

**Published:** 2023-07-19

**Authors:** David Payne, Yongfang Li, Ganesan Govindan, Anuj Kumar, Julie Thomas, Charles A. Addo-Quaye, Andy Pereira, Ramanjulu Sunkar

**Affiliations:** 1 Department of Biochemistry and Molecular Biology, Oklahoma State University, Stillwater, OK 74078, USAyongfang.li@okstate.edu (Y.L.); ganeshmssrf@gmail.com (G.G.); 2 Department of Crop, Soil, and Environmental Sciences, University of Arkansas, Fayetteville, AR 72701, USA; axk018@uark.edu (A.K.); jt008@uark.edu (J.T.); apereira@uark.edu (A.P.); 3 Department of Computer Science and Cybersecurity, Metropolitan State University, Saint Paul, MN 55106, USA

**Keywords:** caryopsis, chalkiness, grain quality, high day temperature stress, microRNAs, rice

## Abstract

High temperature impairs starch biosynthesis in developing rice grains and thereby increases chalkiness, affecting the grain quality. Genome encoded microRNAs (miRNAs) fine-tune target transcript abundances in a spatio-temporal specific manner, and this mode of gene regulation is critical for a myriad of developmental processes as well as stress responses. However, the role of miRNAs in maintaining rice grain quality/chalkiness during high daytime temperature (HDT) stress is relatively unknown. To uncover the role of miRNAs in this process, we used five contrasting rice genotypes (low chalky lines Cyp, Ben, and KB and high chalky lines LaGrue and NB) and compared the miRNA profiles in the R6 stage caryopsis samples from plants subjected to prolonged HDT (from the onset of fertilization through R6 stage of caryopsis development). Our small RNA analysis has identified approximately 744 miRNAs that can be grouped into 291 families. Of these, 186 miRNAs belonging to 103 families are differentially regulated under HDT. Only two miRNAs, Osa-miR444f and Osa-miR1866-5p, were upregulated in all genotypes, implying that the regulations greatly varied between the genotypes. Furthermore, not even a single miRNA was commonly up/down regulated specifically in the three tolerant genotypes. However, three miRNAs (Osa-miR1866-3p, Osa-miR5150-3p and canH-miR9774a,b-3p) were commonly upregulated and onemiRNA (Osa-miR393b-5p) was commonly downregulated specifically in the sensitive genotypes (LaGrue and NB). These observations suggest that few similarities exist within the low chalky or high chalky genotypes, possibly due to high genetic variation. Among the five genotypes used, Cypress and LaGrue are genetically closely related, but exhibit contrasting chalkiness under HDT, and thus, a comparison between them is most relevant. This comparison revealed a general tendency for Cypress to display miRNA regulations that could decrease chalkiness under HDT compared with LaGrue. This study suggests that miRNAs could play an important role in maintaining grain quality in HDT-stressed rice.

## 1. Introduction

Due to increasing population growth, an approximately 70% increase in the food supply will be needed by 2050 to meet the global dietary demands [1]. The Intergovernmental Panel on Climate Change (IPCC) predicts that the mean surface temperature could increase by another 4.8 °C by 2100 [2]. Moreover, heat waves will be more frequent and long lasting than predicted before. The increasing high temperature stress (HS) can affect plant growth and development, consequently decreasing the quality and quantity of grain production.

Rice (*Oryza sativa* L.) is a staple food crop feeding half of the world’s population. HS is known to affect rice growth and development, subsequently decreasing grain yield and quality [3,4,5]. Grain filling is a crucial stage in determining the quality and yield in rice. However, this stage is also the most sensitive to HS. HS negatively impacts rice grains by increasing chalkiness, decreasing grain shape and size, impairing the milling quality of rice, and ultimately affecting the market value of rice [6,7].

Chalkiness refers to the opaque portion found in the otherwise translucent white endosperm. HS has been shown to increase chalk formation by impairing starch biosynthesis, in turn promoting non-uniform grain filling. This results in smaller, irregular and loosely packed starch granules [8,9,10,11]. Gene expression profiles have revealed that starch biosynthesis genes such as starch synthases, starch branching enzymes and sucrose transporters are downregulated while genes encoding starch degrading enzymes such as alpha-amylases are upregulated during HS [12,13,14]. This dysregulation of gene expression ultimately impairs starch accumulation and promotes chalkiness in rice. However, the low chalky genotypes tend to maintain higher expression levels of starch biosynthesis genes under HS when compared to high chalky genotypes [15,16,17]. More recently, it was reported that the lack of coordinated expression of genes associated with starch biosynthesis was a major cause for different degrees of chalkiness in different genotypes [18]. The authors have shown that the transcript levels of ADP-glucose pyrophosphorylase (*AGPase*) peaked prior to the peaking of downstream starch biosynthesis enzymes such as granule bound starch synthase I (*GBSSI*) and starch synthase IIA (*SSIIA*) [18]. This lack of coordination was more severe in high chalky lines [18]. Thus, the extent to which HS affects rice chalkiness differs between the cultivars due to variations in the efficiency of their HS response mechanisms. In the context of HS-induced chalkiness in rice, microRNAs (miRNAs) may be involved in regulating the expression of starch metabolism genes. By incorporating the miRNA analyses during caryopsis development in HS-exposed plants, we could gain a more comprehensive understanding of the gene regulatory networks that are involved in this phenomenon.

Plant miRNAs represent a class of genome-encoded small noncoding RNAs that negatively regulate their target mRNA expression [19,20,21,22]. A majority of conserved plant miRNAs target transcription factors, but few miRNAs could target transporters (sulfate and phosphate transporters) and enzymes associated with reactive oxygen species detoxification and ubiquitination—but rarely genes associated with starch biosynthesis. Thus, a direct link between conserved miRNAs and Gene Regulatory Networks (GRNs) associated with starch metabolism is rather weak, though some of the non-conserved miRNAs appear to target genes of starch metabolism. Direct interactions between non-conserved miRNAs and targets are: Osa-miR1436 and Osa-miR1867 targeting a granule-bound starch synthase (*GBSS*—LOC_Os07g22930) [23], Osa-miR1432 targeting an alpha-amylase (LOC_Os08g36910) [24] and Osa-miR1861 targeting beta-amylase (LOC_Os10g32810) [23]. Osa-miR1428e-3p acts more indirectly by targeting sucrose non-fermenting-1 related kinases (*SnRK1b* and *SnRK1c*—LOC_Os08g37800 and LOC_Os03g17980, respectively) [25]. By phosphorylating, these kinases are known to influencing the activities of many starch biosynthesis enzymes such as SSI, SSIIa, SSIIIa, SBE1 and SBEIIb [26]. On the other hand, an indirect role for miRNAs in starch metabolism could be attributed and these interactions are acting upstream in this pathway. These include miR172, which targets rice starch regulator 1 (*RSR1*) (LOC_Os05g03040), a starch biosynthesis inhibitor [27], miR396 targeting Growth Regulating Factor (*GRF*), which seem to be associated with chalkiness [28], and Osa-miR397 targeting laccases, which promote grain filling [29]. However, the exact mechanisms involved in these indirect interactions are unknown.

While HS responsive miRNAs have not often been studied in the context of grain chalkiness, miRNA profiles in the rice spikelets, which include the caryopsis tissue, have been previously reported. One of these studies has found a relationship between moderate soil drying and increased grain filling in inferior spikelets, most notably involving the downregulation of Osa-miR1432 and the upregulation of Osa-miR397 and Osa-miR1861 [29]. Other studies have suggested that certain miRNAs are associated with either high or low levels of grain filling [29,30,31]. Furthermore, a QTL overlapping with Osa-miR1853 was identified, although how Osa-miR1853 could affect chalkiness was not explored [32].

In this study, we report miRNA profiles specifically from the R6-stage caryopsis tissue and their expression profiles under high daytime temperature (HDT) stress in five genotypes. Importantly, we also compared between “Cypress”, with the genetically closely related “LaGrue”, to better understand miRNA regulations between low chalky and high chalky genotypes under HDT. The observed differential miRNA regulations in different genotypes suggests that the miRNAs could function in promoting or decreasing HDT-induced chalkiness. Overall, this study demonstrated some important links between miRNAs and starch metabolism in the developing caryopsis, thus reflecting the role of miRNAs in affecting the grain quality/chalkiness under HDT. 

## 2. Results

### 2.1. Phenotypic Variation and Trait Correlation Analyses

**Figure 1:**
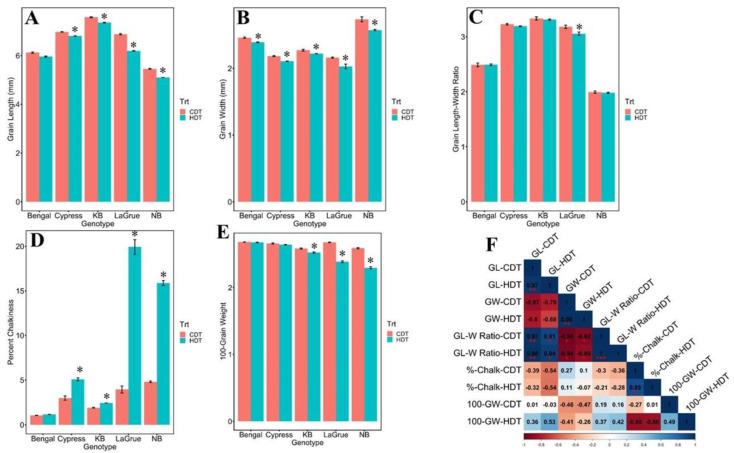
Phenotypic variation in grain quality traits in four Arkansas rice genotypes (Bengal, Cypress, Kaybonnet-KB and LaGrue) and one reference genome cv. Nipponbare (NB) under high daytime temperature (HDT) stress compared to control daytime temperature (CDT) treatment. (**A**) Bar plots showing Grain Length (mm) response to HDT stress compared to CDT treatment. (**B**) Grain Width (mm) response to HDT stress compared to CDT treatment. (**C**) Grain Length–Width Ratio response to HDT stress compared to CDT treatment. (**D**) Percent chalkiness response to HDT stress compared to CDT treatment. (**E**) 100-Grain Wight response to HDT stress compared to CDT treatment. (**F**) Pearson’s correlation coefficient between the grain quality traits (Grain Length-GL, Grain Width-GW, Grain Length-Width Ratio, and percent chalkiness-% chalk) and 100-Grain Weight (100-GW) under CDT and HDT stress treatment. * *p* < 0.05, ** *p* < 0.01, *** *p* < 0.001 indicate significant correlation between the traits.

The effect of HDT on grain phenotypic traits such as Grain Length (GL-mm), Grain Width (GW-mm), Grain Length–Width Ratio (GLW ratio) 100-Grain Weight, (100-GW) and percent chalkiness (% chalk) was assessed in all five rice genotypes. With the exception of GLW ratio, a significant variation was observed for the GL, GW, 100-GW and % chalk under HDT in five genotypes (Figure 1 and Supplementary Figure S1). For GL, LaGrue showed highest reduction (9.92%) followed by Nipponbare (NB) (6.49%), while Cypress exhibited least reduction (2.34%) followed by Bengal (2.62%) and Kaybonnet (KB) (2.91%) under HDT stress (Figure 1A). For GW, LaGrue displayed the most reduction (6.13%) followed by NB (5.95%), while KB showed least reduction (2.3%) followed by Bengal (2.75%) and Cypress (3.40%) under HDT stress (Figure 1B). The GLW ratio was affected in all rice cultivars, and LaGrue was most affected (3.98% reduction) followed by Cypress (1.10%), followed by KB (0.66%) and NB (0.59%) under HDT stress (Figure 1C). The % chalk, a major grain appearance characteristic, was highly affected by HDT stress. LaGrue showed highest increase (80.19%) followed by NB (69.75%), Cypress (41.5%), KB (21.29%) and Bengal (10.07%) (Figure 1D). In 100-GW, a major yield determining component which is also closely linked with % chalk, it showed highest reduction (11.33%) in NB followed by LaGrue (10.83%), while reduction was the lowest in Bengal (0.25%), followed by Cypress (0.69%) and KB (2.32%) under HDT (Figure 1E). This analysis has allowed us to group low chalky genotypes (KB, Cypress, and Bengal) and high chalky genotypes (NB and LaGrue).

The Pearson’s correlation coefficient was used to evaluate the relationship between grain quality traits (GL, GW, GLW ratio and % chalk) and 100-GW under Control Daytime Temperature (CDT) and HDT stress treatment (Figure 1F). Strong correlations were observed between grain quality traits and 100-GW under HDT stress (Figure 1F). Under CDT treatment, 100-GW negatively correlated with % chalk (r = −0.27), while under HDT stress, these negative correlations with % chalk were even stronger (r = −0.86). However, 100-GW was positively correlated with GLW ratio (r = 0.19 and r = 0.42) under CDT and HDT stress treatments, respectively. Like % chalk, 100-GW is negatively correlated with GW (r = −0.46 and r = −0.26) under CDT and HDT stress treatments, respectively, while it is positively correlated with GL (r = 0.01 and r = 0.53) under CDT and HDT stress treatments, respectively.

### 2.2. Summary of sRNA Libraries

**Figure 2:**
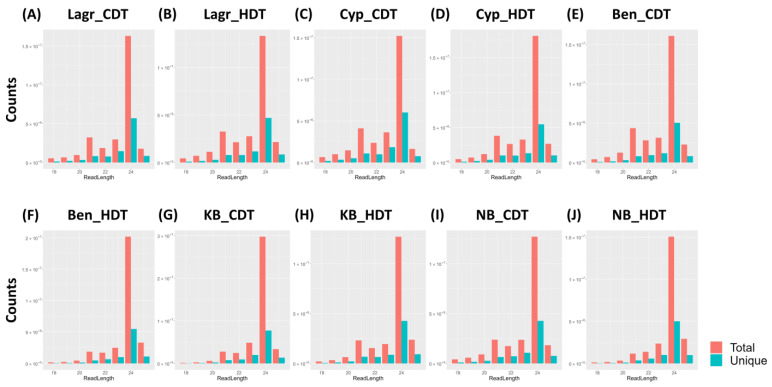
Size distribution of sRNA reads for each genotype under each treatment (CDT and HDT). The number of total and unique reads of each length for the combined three replicates are plotted for (**A**) Lagr under CDT, (**B**) Lagr under HDT, (**C**) Cyp under CDT, (**D**) Cyp under HDT, (**E**) Ben under CDT, (**F**) Ben under HDT, (**G**) KB under CDT, (**H**) KB under HDT, (**I**) NB under CDT and (**J**) NB under HDT.

To determine the effect of heat stress on miRNA profiles in the R6 stage caryopsis samples of five rice genotypes, small RNAs (sRNAs) were sequenced from the CDT and HDT exposed plants. Approximately 388 million total and 130 million unique clean reads were obtained by sequencing a total of 30 sRNA libraries from the caryopsis samples (a total of six libraries for each variety that includes three replicates each from CDT and HDT) (Table S1). The total number of reads ranged between 10,132,746 and 22,959,921 while the unique reads varied between 2,392,228 and 7,769,838 per library. For the individual replicates, between 87.4 and 95.8% of the reads and 76.6 and 93.2% of the unique reads could be mapped to the rice genome (Table S1). These sRNA libraries showed clear peaks for 21-nt and 24-nt reads (Figure 2). A small percentage of the reads (0.6 to 2.7% of the clean reads and 0.2–0.4% of the unique reads) were mapped to rice miRNA precursors available at the miRBase (Table S1). Most of these observed features are consistent with typical plant sRNA libraries [33,34,35,36,37,38].

### 2.3. Identification of Novel miRNAs

In general, the spatial and temporal regulation of miRNAs varies between different tissues or organs of rice [39,40,41]. Thus far, a few studies have analyzed miRNAs in rice developing grains [42,43,44], while other studies used entire spikelets [45,46]. In this study, we specifically used caryopsis tissue from the R6 stage, which is defined as “the grain depth expansion stage at which milky starch in the developing grain is beginning to become firm but is still soft” [47], for analyzing miRNAs regulated by HDT. Given that we sequenced 30 libraries from R6 stage caryopsis tissue, there is a potential for finding novel miRNAs. After thoroughly scrutinizing for abundance, consistent foldback structures, and processing precision, 14 potential novel miRNA loci were identified using ShortStack [48] (Table 1 and Table S2, Figure S2). Among them, four MIRNA (canH-MIR9774b, canN-01, canN-08, canN-09) loci were within intergenic regions, six were within introns (canH-MIR11339, canN-02, canN-03, canN-04, canN-05, canN-06), two loci (canH-MIR2905, canN-07) were on the opposite strands of intronic sequences, one in the 3′ UTR of a gene (canH-MIR9774a) and one (canH-MIR169 was originated from the opposite strand of Osa-MIR169p (Chr8:7,641,327-7,641,452 [-] in MSU7).

The newly identified miRNAs belonging to known families were labeled as “canH” miRNAs and identified by their family number, while novel miRNAs were labeled as “canN” miRNAs. The 24-nt long canH-miR2905-3p and canH-miR11339-5p aligned with two mismatches to the known 21-nt sequences Osa-miR2905 and Osa-miR11339, respectively (Figure S3). Similarly, the identical 23-nt canH-miR9774a-3p and canH-miR9774b-3p aligned to the 22-nt Tae-miR9774 with one mismatch (Figure S3). Finally, the canH-miR169-5p matches with Osa-miR169c with a 1-nt shift (Figure S3). The remaining nine loci represent putative novel miRNAs, eight of which were recovered from all five genotypes. The only exception was canN-07, which was not recovered from Bengal. One of the novel miRNAs, canN-01, has a homologous sequence in *Brachypodium distachyon* with convincing hairpin structure, suggesting that it is likely conserved in another monocot (Figure S2). The other eight novel miRNAs seemed to be specific to rice.

### 2.4. Known miRNAs and Their Abundances in R6 Stage Caryopsis Samples

By mapping the unique reads to miRBase (www.mirbase.com), we have identified a total of 726 known miRNAs (5p and 3p miRNAs) belonging to 282 miRNA families (Tables S3 and S4). To assess their overall abundances in R6 caryopsis tissue, the normalized reads (Counts Per Million clean reads—CPM) for each library were used. In general, the highly conserved miRNA families were relatively abundant in R6 caryopsis tissue, with the exception of miR395, miR398 and miR399, whose abundances were low (Table S4). This is expected because these three miRNAs are expressed at very low abundance under normal conditions but were induced only during specific nutrient deprivations [22,49,50]. The most abundant miRNAs include Osa-miR166a-d,f,j-3p, Osa-miR1874-3p, Osa-miR1425-5p, Osa-miR396e-5p, Osa-miR1866-5p, Osa-miR167d-j-5p, Osa-miR1850.1*, Osa-miR1876, Osa-miR156a-j-5p, Osa-miR1877, Osa-miR166m and Osa-miR444b.1_c.1. Interestingly, two of the newly identified miRNAs (canN-06-5p and canH-miR9774ab-3p) are also highly abundant in R6 caryopsis samples (Table S4).

Typically, miRNAs are more abundantly expressed than their miRNA-star sequences. However, several studies have reported that in some cases, the miRNA* levels could be as abundant, or even more abundant, than their corresponding miRNA levels [51,52]. In the current study, Osa-miR159a.2, Osa-miR159f, Osa-miR398b, Osa-miR529a, Osa-miR810a, Osa-miR810b.1, Osa-miR1430, Osa-miR1850.1, Osa-miR1855, Osa-miR1873, Osa-miR5155, Osa-miR5339, Osa-miR5510 and several members of the Osa-miR169 family, the miRNA* levels were considerably more abundant than their corresponding miRNAs (Table S4).

We did not recover a few previously reported rice miRNAs from the R6 samples of caryopsis of specific genotypes. These include Osa-miR2090, which was not found in Ben, KB, or LaGrue, Osa-miR3980a and Osa-miR3980b, which were not detected in NB, Osa-miR5155, which was not detected in KB samples, and the novel miRNA canN-07, which was absent in Bengal samples (Table S4). It is possible that these miRNAs are not expressed in this specific tissue of particular genotypes or that the abundances of these miRNAs at R6 stage is extremely low in these genotypes.

### 2.5. Differential MicroRNA Expression under HDT in Low Chalky and High Chalky Lines

**Figure 3:**
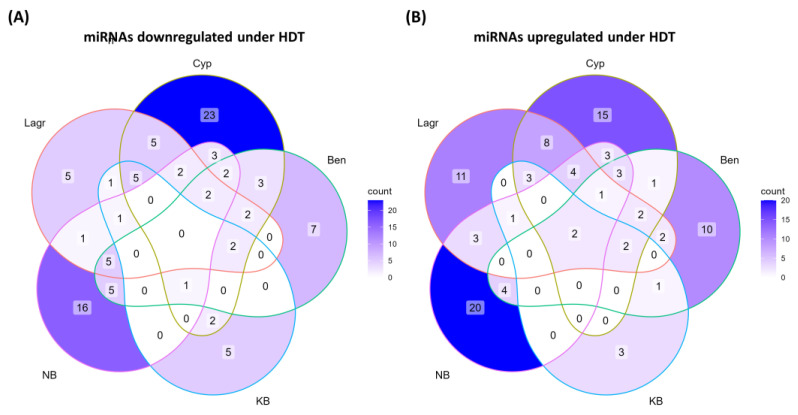
Venn diagram depicting differences in miRNA regulation under HDT in five genotypes. (**A**) downregulated or (**B**) upregulated miRNAs.

To gain an insight into miRNA alterations in R6 caryopsis tissue exposed to HDT, EdgeR was used to assess the HDT vs. CDT comparison in each genotype with a significance cutoff of *p* value < 0.05. This analysis has identified a total of 186 differentially regulated miRNAs belonging to 103 miRNA families (including 6 novel miRNA families) in all of these genotypes (Figure 3 and Figures S4–S8, Tables S5–S9). Notably, several of these sequences are miRNA-star sequences. Overall, Cypress had a maximum number of differentially regulated miRNAs (96) of which 44 and 52 were upregulated and downregulated, respectively. By contrast, only 29 differentially regulated miRNAs (12 upregulated and 17 downregulated) were identified in KB. Remarkably, only two miRNAs (Osa-miR444f and Osa-miR1866-5p) were commonly upregulated in all five genotypes under HDT (Figure 3 and Figures S4–S8, Table S10). Furthermore, none of the miRNAs were commonly downregulated in all five genotypes.

#### 2.5.1. Shared Upregulations among the Low Chalky and High Chalky Genotypes

On the basis of percent chalkiness under HDT (Figure 1D), genotypes were categorized into the high chalky group (LaGrue and NB) and the low chalky group (Cypress, Bengal, and KB). Only two commonly upregulated miRNAs (Osa-miR444f and Osa-miR1866-5p) were identified in all five genotypes (Table S10).

While there are only few shared upregulated miRNAs among all three low chalky genotypes, the miRNAs that are upregulated in at least two of the three low chalky genotypes (Cypress, Bengal, KB) may be of importance for HDT tolerance. These include seven miRNAs which were upregulated in both Cypress and Bengal, three miRNAs (Osa-miR164e, Osa-miR164e* and Osa-miR167e,i-3p) which were upregulated in Cypress and KB, and Osa-miR1876*, which showed shared upregulation between Bengal and KB. However, it should be noted that all of the above upregulated miRNAs besides Osa-miR1876* were also upregulated in at least one of the two high chalky genotypes, thus it is hard to attach importance to these miRNAs from this analysis.

Three upregulated miRNAs (Osa-miR1866-3p, Osa-miR5150-3p and canH-miR9774ab-3p) were shared between two high chalky genotypes (LaGrue and NB) and none of the low chalky genotypes, suggesting that the similarities between the high chalky genotypes are also low.

#### 2.5.2. HDT-Upregulated miRNAs Are Largely Genotype-Specific

Remarkably, a total of 59 upregulated miRNAs were genotype-specific. For instance, 20 miRNAs were upregulated only in NB, 15 miRNAs upregulated only in Cypress, 11 miRNAs upregulated only in LaGrue, 10 miRNAs upregulated only in Bengal and 3 miRNAs upregulated in KB (Table S10).

#### 2.5.3. Shared Downregulations among the Low Chalky and High Chalky Genotypes

There are at least three miRNAs (Osa-miR1428e,f,g, Osa-miR169r-3p and Osa-miR2121a*) whose levels were downregulated in all three low chalky genotypes. However, each of these were also downregulated in either LaGrue or NB. Thus, these were not specific to the low chalky group alone (Table S10).

Other downregulated miRNAs that were shared between at least two of the three low chalky genotypes (Cypress, Bengal and KB) include nine miRNAs which were commonly downregulated between Cypress and Bengal and seven miRNAs which displayed shared downregulation in Cypress and KB. Several of these miRNAs were also downregulated at least in one of the two high chalky genotypes, although canH-miR169-3p, Osa-miR166e-3p, Osa-miR319a-3p, Osa-miR3980a,b-3p and Osa-miR5339* were exceptions.

Eleven miRNAs were commonly downregulated in LaGrue and NB, the high chalky genotypes. However, most of these miRNAs were also downregulated in at least one of the low chalky genotypes, thus these regulations are not unique to high chalky genotypes. The only miRNA that was downregulated by HDT in the high chalky genotypes, LaGrue and NB, was Osa-miR393b-5p.

#### 2.5.4. Shared Downregulations among the Low Chalky and High Chalky Genotypes

Reminiscent of the upregulated miRNAs, a significant number of downregulated miRNAs (56) were genotype-specific. These include 23 miRNAs downregulated only in Cypress, 16 miRNAs downregulated only in NB, 7 miRNAs downregulated only in Bengal, 5 miRNAs downregulated only in KB and 5 miRNAs downregulated only in LaGrue (Table S10). Taken together, these findings imply that the miRNA downregulations under HDT were largely genotype-specific.

### 2.6. Comparison of HDT-Responsive miRNAs between Genetically Closely Related Cypress (Low Chalky) and LaGrue (High Chalky) and Their Validations

**Figure 4:**
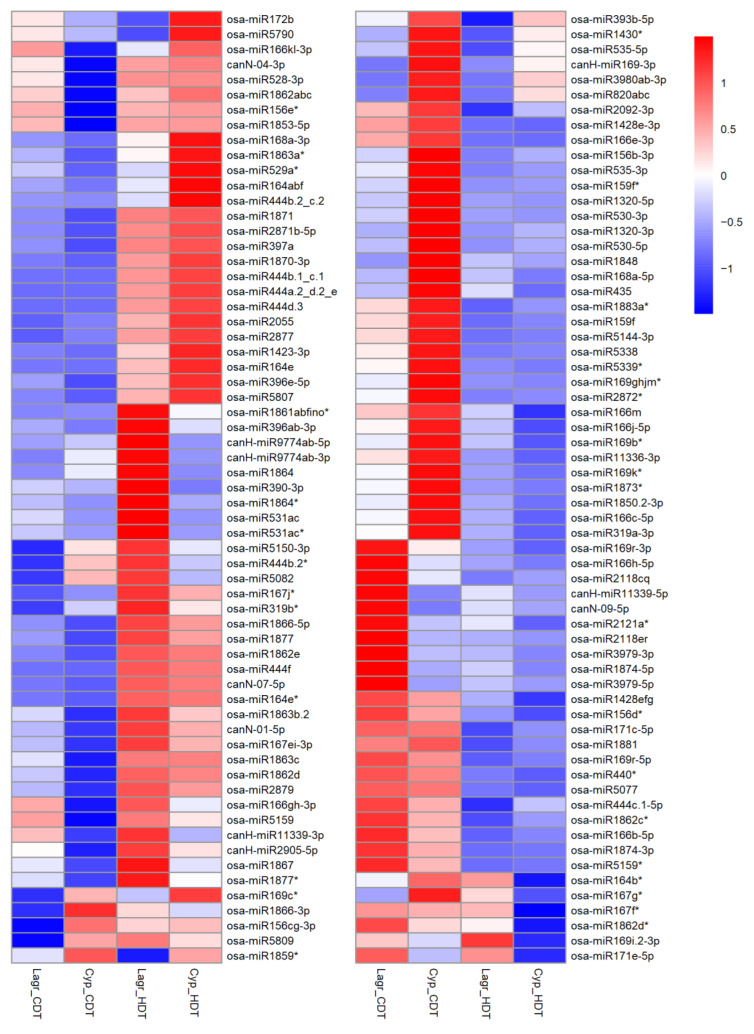
Heatmap (z-scores) displaying miRNAs with significant differential expression in the LaGrue and Cypress. ‘*’ followed by miRNA annotation denotes miRNA star sequences.

Genetically, the Cypress and LaGrue are closely related relative to the other genotypes analyzed in this study, yet they display contrasting chalkiness under HDT. Therefore, comparing HDT responses between these two genotypes may be particularly insightful. A total of 126 miRNAs belonging to 72 miRNA families were significantly regulated in one or both of these genotypes (Figure 4, Figures S4 and S5, Tables S5 and S6).

Several miRNAs showed similar regulation in both genotypes, i.e., either upregulation or downreguation under HDT, but the degree of their regulation greatly differed between LaGrue and Cypress. For instance, four miR444 family members, Osa-miR164e, Osa-miR396e-5p and Osa-miR397a levels were upregulated in both genotypes, although the fold change is higher in Cypress in each case (Tables S5 and S6).

Several miRNAs were upregulated or downregulated in Cypress while being unaltered in LaGrue under HDT (Tables S5, S6 and S10). Among these the most important miRNAs are the upregulated Osa-miR164a,b,f, Osa-miR166k,l-3p, Osa-miR172b and Osa-miR444b.2.-c.2, and the downregulated Osa-miR319a-3p, Osa-miR528-3p (a miRNA*), Osa-miR535-5p and Osa-miR820a,b,c miRNAs. It is also notable that Osa-miR390-3p (a miRNA*) and Osa-miR393b-5p were upregulated and downregulated, respectively, in LaGrue, but not in Cypress. Furthermore, miR166 and miR169 family members found to be downregulated more often in Cypress than in LaGrue under HDT. These differences between Cypress and LaGrue, which are genetically related but differ in their sensitivities to HS, may imply a role for miRNAs in maintaining grain quality (low chalkiness) under HDT, but revealing the mechanisms needs further in-depth studies.

**Figure 5:**
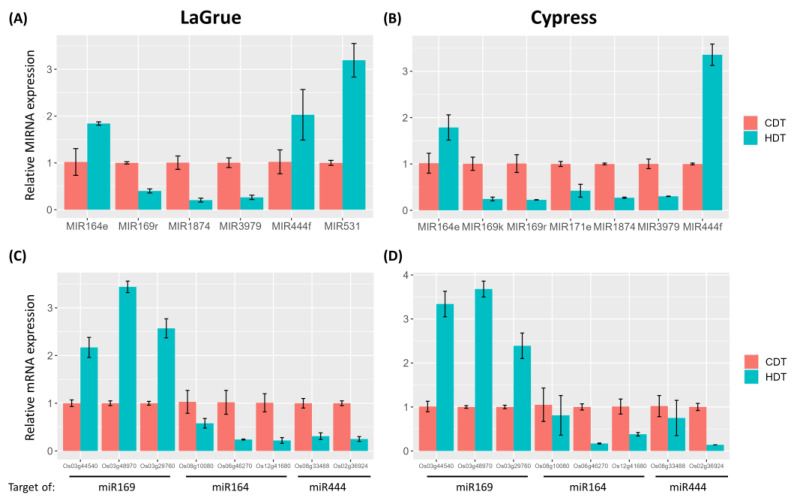
Validation of differentially expressed miRNAs (**A**,**B**) and target mRNAs (**C**,**D**) using quantitative RT-PCR analysis. (**A**,**C**) LaGrue; (**B**,**D**) Cypress. U6 snRNA and actin-2 were used as the reference genes to normalize miRNA precursor and target mRNA expression, respectively. Expression levels under CDT were set as 1, and the error bars represent the standard deviation (SD) of three replicates.

To validate HDT-induced differentially regulated miRNA profiles in LaGrue and Cypress, miRNAs that showed similar or distinct HDT responses in these genotypes were selected (Table 2). For instance, Osa-MIR169r, Osa-MIR1874 and Osa-MIR3979 were commonly downregulated in LaGrue and Cypress, while OsaMIR164e and Osa-MIR444f were commonly upregulated (Figure 5A,B). These expression profiles were confirmed using qRT-PCR in both genotypes. Likewise, the upregulation of Osa-MIR531 was confirmed in LaGrue (Figure 5A), whereas the upregulation of Osa-MIR164e and the downregulation of Osa-MIR169k and Osa-MIR171e were validated in Cypress (Figure 5B). These results suggest that the sRNA sequencing profiles were largely reliable. We also checked the expression profiles of several target genes of miR164, miR169 and miR444 in HDT-treated LaGrue and Cypress. As expected, the target genes displayed opposite regulation patterns to the miRNA profiles (Figure 5C,D).

### 2.7. Comparison of miRNA Levels between Genetically Closely Related Cypress (Low Chalky) and LaGrue (High Chalky)

Additional differential expression analyses were performed to determine the relative miRNA expression values between Cypress and LaGrue under CDT (Cyp_CDT vs. Lagr_CDT) and HDT (Cyp_HDT vs. Lagr_HDT). Notably, genotype-specific differences are evident even under control (CDT) conditions. Under CDT, 32 and 40 miRNAs showed significantly higher abundances (the normalized read counts are significantly higher in Cyp control samples) and lower abundances (the normalized read counts are significantly lower in Cypress), respectively, in control samples of Cypress compared to LaGrue (Table S11, Figure S9A). Under HDT, 34 miRNAs were more abundant in Cypress than in LaGrue, (the normalized values are significantly higher in HDT-treated Cypress than in Lagr_HDT) and 42 were more abundant in LaGrue than Cypress (the normalized values are significantly higher in LaGrue compared to Cypress under HDT) (Table S12, Figure S9B).

Interestingly, Osa-miR166c-5p, Osa-miR167g*, Osa-miR169b* and Osa-miR169k* levels were more abundant in Cypress than in LaGrue under CDT, but their levels were more abundant under HDT in LaGrue, i.e., the normalized values were significantly higher in Cyp_CDT than Lagr_CDT, but these values are significantly higher in Lagr_HDT than Cyp_HDT (Tables S11 and S12), suggesting that these are genotype-specific regulations occurring only under HDT. However, since these are all annotated as miRNA*, it is unknown whether these regulation patterns are important or not.

### 2.8. Analysis of the Interaction Term

An interaction term analysis was performed to determine significant differences between Cyp and Lagr under HDT. For about 23 miRNAs the (Cyp_HDT—Cyp_CDT) value was significantly higher than the (Lagr_HDT—Lagr_CDT) value, and for 28 miRNAs, the (Cyp_HDT—Cyp_CDT) value was significantly lower than the (Lagr_HDT—Lagr_CDT) value (Table S13). This analysis offers further confidence in several of the reported results in this study. Notable miRNAs having significantly higher (Cyp_HDT—Cyp_CDT) values in Cypress than in LaGrue (Lagr_HDT—Lagr_CDT) include Osa-miR164abf, Osa-miR172b, Osa-miR166kl-3p, Osa-miR396e-5p and Osa-miR444b.2_c.2, the second most abundant miR444 isoform. Notably, Osa-miR172b and Osa-miR396e-5p could play a role in decreasing chalkiness under HDT [27,28]. These results support our findings that Osa-miR164abf, Osa-miR172b, Osa-miR166kl-3p and Osa-miR444b.2_c.2 were significantly upregulated in Cypress, but not in LaGrue. While Osa-miR396e-5p was found to be significantly upregulated in both genotypes, the interaction term analysis suggests that the upregulation was significantly greater in Cypress. On the other hand, Osa-miR168a-5p, which targets Argonaute, had a significantly lower (Cyp_HDT—Cyp_CDT) value in Cypress than in LaGrue (Lagr_HDT—Lagr_CDT), corroborating our findings that it is significantly downregulated by HDT only in Cypress.

## 3. Discussion

The grain quality analysis (GL, GW and GLW ratio, 100-GW and % chalk) revealed that these attributes were negatively impacted by HDT stress in all five rice genotypes (Figure 1), suggesting that HDT stress impairs grain development, and consequently, the grain weight. Our analysis of 100-GW has shown a strong negative relationship with % chalk under HDT stress, which is consistent with a previous report [53]. Reduced single-grain weight in heat-stressed plants often co-occurs with the reduced activity of starch metabolism enzymes such as sucrose synthase, starch synthase and various invertases [53].

Elevated temperature either during daytime or nighttime during grain filling compromises rice grain quality by promoting chalkiness. The chalky grains result from the disruption of GRNs chiefly associated with starch biosynthesis in the caryopsis during the grain filling process. Previous analyses have shown that genotypes that maintain high starch biosynthesis or low starch breakdown under heat maintain better grain quality coupled with display less chalkiness [15,16,17,54]. For analyzing the miRNAs regulated by HDT, we specifically used R6 stage caryopsis tissue (developing caryopsis) in this study [47,55]. Though some previous studies have characterized heat responsive miRNAs in developing grains or spikelets, this study is noteworthy in several respects: (1) the caryopsis at R6 stage of rice grain development was used for examining the effects of HDT on miRNA profiles; (2) five rice genotypes with varying levels of grain chalkiness under HDT, including two genotypes that are genetically closely related yet display contrasting chalkiness (LaGrue and Cypress), were used for drawing meaningful comparisons; and (3) a longer duration (immediately after pollination through R6 stage) of HDT was used to better mimic field conditions than the short duration heat stress, which was often used in heat stress studies. 

Remarkably, a total of 744 miRNAs belonging to 282 known miRNA families were detected in this minute tissue of R6 stage caryopsis sample. Interestingly, almost all known conserved rice miRNAs and majority of the non-conserved miRNAs were detected in R6 caryopsis tissue. This study also allowed us to confidently identify 9 novel miRNAs in this tissue given that we sequenced 30 small RNA libraries from 5 different genotypes. Consistent with many plant studies including rice, the abundances of conserved miRNA families were far greater than that of non-conserved and novel miRNAs. The higher abundances of conserved miRNAs suggest that miRNA-controlled gene regulation is ubiquitous in this specific stage of caryopsis tissue, thus revealing the role of miRNAs in starch and protein metabolism in developing rice grains. There are also some rice-specific miRNAs (Osa-miR1874-3p, Osa-miR1425-5p, Osa-miR1866-5p, Osa-miR1850.1*, Osa-miR1876 and Osa-miR1877) that are very highly expressed in this tissue (Table S4). On the basis of their abundant accumulation in the caryopsis and their differential regulation under HDT, the involvement of miRNAs in grain filling and HDT response is apparent.

### 3.1. Only Two miRNAs Displayed Shared Regulations among All Five Genotypes under HDT

Overall, the differential miRNA regulation in response to HDT had little overlap among the five rice japonica genotypes. Only Osa-miR1866-5p and Osa-miR444f were upregulated in all of these genotypes, but none were downregulated in all of them. Though the precise role is unknown, the upregulation of Osa-miR1866 has been positively associated with grain filling [29]. Not only was Osa-miR444f upregulated in all five genotypes, but another member of this family, Osa-miR444a.2_d.2_e, was also upregulated in all genotypes except in NB. Furthermore, Osa-miR444d.3 was upregulated in Lagrue, Cyp and Ben. These observations largely implicate a role for miR444 family in caryopsis development under HDT via its targets, MADS box transcription factors. The shared upregulation of these two miRNA families (Osa-miR444 and Osa-miR1866-5p) in both low chalky and high chalky lines suggests that these miRNAs could be part of a general heat stress response mechanism in R6 caryopsis tissue. Indeed, miR444 has been shown to be upregulated in response to heat stress in maize [56].

### 3.2. HDT-Responsive miRNAs Are Largely Genotype-Specific

The majority of differentially upregulated or downregulated miRNAs under HDT are largely genotype-specific (Figure 3). The genotype-specific miRNA regulations have been reported in cotton [57] and rice [58] under salt stress, cowpea under drought [59], wheat under heat stress [60,61], and in rice under short and prolonged heat stress [62], though not specifically in the caryopsis samples.

### 3.3. Differential Regulation of Highly Conserved miRNAs under HDT Suggests an Indirect Role in Grain Filling

Making direct connections between miRNA regulation in developing grains and HDT-induced chalkiness is difficult because of the lack of substantial evidence linking the known/predicted miRNA targets that are directly involved in starch metabolism in general. A few direct links are through miR1436 and miR1867, both of which could target *GBSSII* [23], as well as miR1432 and miR1861, which could target alpha [24] and beta [23] amylases, respectively. Additionally, miR1861 targets a starch synthase inhibitor (LOC_Os01g63810) [29,63]. The HDT-regulated miRNA, Osa-miR1428e-3p could affect starch metabolism indirectly by inhibiting SnRK1b and SnRK1c, which are kinases known to influence (activate/suppress) the activity of several enzymes in the starch biosynthesis pathway [26]. Therefore, Osa-miR1428e-3p could influence starch biosynthesis and forms a major indirect link between HDT-regulated miRNAs and chalkiness.

Downregulation of miR159 by short-term heat stress (one day or less) has been reported in rice [64,65], wheat [66,67] and in the long-term heat stress (5 days) in maize [68]. Our observation of downregulation of at least one isoform of Osa-miR159 in all genotypes (with the exception of KB) is consistent with these previous reports. It is unknown whether the HS-induced downregulation of Osa-miR159 in the caryopsis could be a protective mechanism against HS-induced grain chalkiness. Osa-miR159 downregulation is likely to upregulate its targets, MYB transcription factors, which in turn could regulate many downstream genes important for grain quality under HDT. Indeed, overexpression of miR159 in rice resulted in increased sensitivity to heat-stress [66], suggesting that the downregulation of miR159 is beneficial under heat stress. Consistent with this, it was shown that Osa-miR159 is negatively associated with grain filling [29], although another study found more ambiguous results [30]. Taken together, we suggest an important role for the downregulated miR159 in developing caryopsis samples under HDT because this miRNA was similarly regulated in majority of the genotypes studied here.

Short-term heat stress (less than 2 days) has been shown to upregulate miR166 expression in rice [64,65,69], barley [70] and wheat [71]. Two isoforms of miR166 (Osa-miR166g,h-3p and Osa-miR166k,l-3p) were upregulated in Cypress (low chalky line), while two other isoforms (Osa-miR166e-3p and Osa-miR166m) were downregulated. The somewhat conflicting observations between our results and the previous studies could be due to the longer duration of heat stress used in our study. The miR166 family targets bZIP transcription factors, and OsbZIP58/RISBZ1 (LOC_Os07g08420) was shown to be negatively associated with chalkiness [72], though it is not a target for the Osa-miR166 family. However, it is possible that other bZIPs, including those targeted by miR166, could play a role as reported earlier [72]. 

miR169 isoforms were shown to be downregulated under short-term (1 day) heat stress in rice [64], and longer-term (5–7 days) heat stress in wheat [61], maize [68] and Arabidopsis [73]. Consistent with this, at least one isoform of Osa-miR169 was downregulated by HDT in all genotypes except in NB. The miR169 family targets *NF-YA* subunits, which form trimeric NF-Y transcription factors along with the NF-YB and NF-YC subunits. Some of the NF-YB and NF-YC subunits are involved in promoting grain development and preventing chalkiness [74,75,76,77,78], but whether we can attribute a similar role for *NF-YA* subunits targeted by the miR169 family in this study is unclear.

Osa-miR393b-5p, which targets F-box genes, was downregulated in the LaGrue and NB (both high chalky lines) under HDT. The miR393 is known to target F-box proteins (E3 ligases including *TIR1*, an auxin receptor), and it is unclear if or how this may affect grain quality under heat stress. Nevertheless, the observation that miR393 levels were downregulated in both of the high chalky lines, but none of the low chalky lines, suggests a potential role for auxin signaling in maintaining grain quality under HDT. In fact, previous studies have implicated a role for auxin signaling in grain development and that miR393 may have a role in this process [79,80].

Osa-miR397a levels were upregulated in Cypress and LaGrue, and this increase was greater in Cyp (2.25-fold change) than in Lagr (1.30-fold change). The miR397 family, which targets laccase genes, was shown to be positively associated with starch biosynthesis [29]. The higher magnitude of Osa-miR397a upregulation under HDT in Cypress compared to LaGrue could positively contribute to low chalkiness.

High temperature is known to induce oxidative stress; therefore, redox-responsive miRNAs could be part of HDT-responsive GRNs. At least four conserved miRNAs (miR397, miR398, miR408 and miR528) have been implicated in redox responses in plants [81,82]. However, our analysis did not reveal major regulations of these miRNAs under HDT. Interestingly, two miRNA-stars of these miRNAs, miR398a* and miR528-3p (miR528*), were significantly elevated under HDT in NB and Cyp, respectively, but their functions remain unclear.

### 3.4. MicroRNA Profiles Differed between Genetically Closely Related Cypress (Low Chalky Line) and LaGrue (High Chalky Line)

Osa-miR164a,b,f levels were significantly more upregulated by HDT in Cypress than in LaGrue. This observation is consistent with the report that the Osa-miR164 is positively associated with grain filling [30]. The upregulated 164 is expected to decrease the expression of *NAC* TFs, and this module could play a positive role in maintaining grain quality and decrease chalkiness under HDT.

The miR172 family targets *APETALA2* members, including *RSR1* (LOC_Os05g03040), which negatively regulates type I starch synthesis genes [27]. Osa-miR172b levels were significantly upregulated by HDT in Cypress, but not in LaGrue. The HDT-elevated miR172 could inhibit RSR1 to a greater extent in Cypress, thus contributing to better grain filling under HDT in this genotype.

Osa-miR1428e-3p plays an important role in starch metabolism by targeting *SnRK1b* and *SnRK1c* [25,83]. SnRK1 phosphorylates, which in turn increases activity of several enzymes of starch biosynthesis, i.e., sucrose synthase (SUS) [26,84,85,86], invertase (INV) [26], UDP-glucose pyrophosphorylase [26], ADP-glucose pyrophosphorylase (AGPase) [26,85,86], starch branching enzyme (*SBE*) [26] and starch synthase (SS) [26,85]. Contrastingly, it inhibits sucrose phosphate synthase (SPS) by phosphorylation [85,87]. It is possible that SnRK1 may directly phosphorylate SUS to increase its activity [88,89,90,91]. However, SnRK1 is not likely to strongly affect AGPase through direct phosphorylation [92], but it may increase AGPase activity by altering its redox state [93]. Due to the positive role of SnRK1 in starch accumulation, we expect Osa-miR1428e-3p to be associated with high chalkiness. Surprisingly, osa-miR1428e-3p was significantly downregulated by HDT in not only Cypress, but also in the high chalky LaGrue and NB genotypes. Furthermore, the log2FC value in NB (−2.29) was much larger than that in Cypress (−1.23), although LaGrue had a smaller log2FC value (−0.88) than Cypress.

Osa-miR1432-5p has been suggested to target an alpha-amylase [24], one of the genes most directly contributing to chalkiness through the degradation of starch [94]. However, another study suggested that Osa-miR1432 inhibits grain quality [80]. In this study, Osa-miR1432-5p had higher expression levels in Cypress than LaGrue under HDT, suggesting that this could inhibit starch degradation and contribute to low chalkiness.

Osa-miR1848 is likely to decrease grain quality by targeting obtusifoliol 14α-demethylase (LOC_Os05g12040), a key enzyme for the synthesis of brassinosteroids, which promote grain filling [95]. Osa-miR1848 was suppressed by HDT in Cypress, possibly contributing to the maintenance of Cypress grain quality under HS. Osa-miR1846, Osa-miR1855, Osa-miR1858 and Osa-miR1859, which are positively associated with grain filling [30], were expressed at higher levels in Cypress under both CDT and HDT. This is especially notable for Osa-miR1855, which was not detected in LaGrue at all. Osa-miR1846, which has been found to be highly expressed during grain filling in the spikelets [46], has been shown to target a heat shock factor (*HSF*) [96]. HSFs may induce chalkiness by promoting heat shock protein (HSP) expression [8,97]. Therefore, it is possible that higher Osa-miR1846 levels in the Cypress repress this HSF, in turn decreasing chalkiness.

Osa-miR1867 targets *GBSSII* (LOC_Os07g22930) [23], an enzyme involved in starch biosynthesis. Osa-miR1867 levels were upregulated by HDT in Cypress, LaGrue and NB. While this observation makes sense for the high chalky LaGrue and NB genotypes, it is unexpected for Cypress. However, it is worth noting that the log2FC is lower in Cypress (0.53) than in LaGrue (0.63) or NB (0.87). Additionally, Osa-miR1867 had significantly higher expression in LaGrue than Cypress under both CDT and HDT, suggesting that this regulation could decrease the starch synthesis rate, leading to high chalkiness.

Osa-miR5144-3p could affect protein accumulation through targeting a protein disulfide isomerase-like (*PDIL*) gene (LOC_Os11g09280) [98], an essential protein folding factor that prevents chalkiness in grains [98,99]. Osa-miR5144-3p was downregulated by HDT in the Cypress and LaGrue genotypes. Nevertheless, the fold change (log2FC) was higher in Cypress (−2.29) than in LaGrue (−1.63), which in turn could be interpreted as a positive regulatory mechanism in Cypress.

These above-described observations suggest that mild differences exist between the miRNA regulatory patterns of HDT-stressed Cypress and LaGrue. Several miRNAs tend to be regulated similarly in both genotypes, but the degree of induction/suppression often varied between the genotypes. Overall, the HDT-induced downregulation of miRNAs inhibiting starch accumulation and upregulation of miRNAs promoting starch accumulation tends to be more pronounced in Cypress. Whether these apparent variations contribute to the better grain quality maintenance of Cypress needs further studies.

Taken together, several conserved and non-conserved miRNAs including rice-specific miRNAs are differentially regulated under HDT in rice genotypes. When compared between the genetically related genotypes Cypress and LaGure, the majority of these regulations differed between low chalky Cypress compared with high chalky LaGrue. Thus, this overall current analysis has offered insights into the differences in miRNA-dependent post-transcriptional gene regulations in R6-stage caryopsis samples under HDT.

### 3.5. HDT-Responsive miRNA Stars in Rice Caryopsis Tissue

Interestingly, several miRNA-stars are upregulated or downregulated under HDT (Tables S5–S9, Figures S4–S8). The earlier notion was that these star molecules are non-functional, but in the past decade, several reports suggested an important role for them because they are dynamically regulated in a tissue-specific manner [52,100,101]. Similarly, some of the miRNA-star expression levels differed greatly under stress. For example, some were upregulated (miR408 and miR482) while some others were downregulated (miR159d-5p, miR171b-5p, miR172b,h,I,j-5p, miR390a,c-3p, miR394a-3p and miR1507c-5p) in response to water stress in soybean root tips, and these regulations were not dependent on their corresponding miRNA levels [36]. In the current study, we have identified differential regulation of several miRNA-stars in the HDT-exposed caryopsis samples (Tables S5–S13, Figures S4–S8). In fact, heat stress has been shown to differentially regulate 15 miRNA stars in maize tissues including reproductive tissues [56]. Studies have also shown that miRNA-stars can regulate target genes [102,103,104]. For instance, miR393-star targets a MEMB12 transcripts encoding Golgi-localized SDS-resistant soluble N-ethylmaleimide sensitive factor attachment protein receptor (SNARE) protein [103]. Thus, our finding of differential regulation of several miRNA-stars under HDT in rice developing grains suggests potentially important roles for miRNA-stars also in grain quality/chalkiness.

## 4. Materials and Methods

### 4.1. Plant Material and Growth Conditions

Four elite Arkansas rice genotypes (Bengal, Cypress, Kaybonnet-KB and LaGrue), along with the Nipponbare (NB), were used in this study. After breaking the seed dormancy, 30 seeds from each genotype were germinated in single plastic pots (15 cm × 15 cm) filled with a 3:1 mix of the Sun Gro professional potting mix (Sun Gro Horticulture Distribution, Agawam, MA, USA) and field soil and grown in the greenhouse at the University of Arkansas, Fayetteville, AR, USA [7,105]. After 10 days of germination, equal-sized seedlings of each genotype were transplanted to 3-gallon plastic pots filled with the mixture of potting mix and field soil and grown in the greenhouse until flowering. The temperature in the green house was maintained at 30 ± 1 °C (86 ± 1 °F) during the day and 22.2 ± 1 °C (72 ± 1 °F) at night [7,106] with a light/dark 13/11-h cycle with maximum photosynthetically active radiant (800–1000 μmol PAR m^−2^ s^−1^) light and 60–65% relative humidity (RH). A completely randomized design (CRD) with three replications (each replication is one plant in the pot) was used. The pots were monitored for water content daily and fertilized with the Peter Professional soluble fertilizer (Allentown, PA, USA) containing chelated iron once a week for full vegetative growth.

### 4.2. Phenotyping, Stress Treatment and Tissue Collection

Immediately after anthesis and 45 min before pollination [107], three main panicles per plant were tagged and the spikelets per panicle in each plant were marked after pollination. These plants were then transferred to the growth chambers maintained at high daytime temperature (HDT—day/night temperature of 38 °C (100.4 °F)/22 °C (71.6 °F) for 6 h (9:00 h–15:00 h), while the control daytime temperature (CDT) treatment was set at a day/night temperature of 30 °C (86 °F)/22.2 °C (72 °F) until harvest maturity (approximately 18–20% grain moisture content). For small RNA analysis, the spikelets with R6 stage (soft milking stage) caryopsis were collected and immediately frozen in liquid nitrogen. The CDT and HDT treatments, including the day and nighttime temperatures, were monitored by installing the HOBO data loggers/sensors (Onset HOBO^®^ data logger, Cape Cod, MA, USA) until physiological maturity. The data logger system was operated by the HOBOware^®^ Pro software (Version 1.12.1)/app with compatible devices. At harvesting maturity, the tagged panicles (under CDT and HDT stress treatments) for each genotype were collected separately in individual brown bags, air-dried (12–14% grain moisture content), and used for the phenotyping of grain quality traits such as grain length (GL-mm), grain width (GW-mm), grain length–width ratio (GLW ratio), percent chalkiness (% chalk) and 100-grain weight (100-GW).

For grain quality traits estimation, rough rice was de-hulled and GL (mm), GW (mm) and GLW ratio, and % chalk (expressed as percent of affected in the projected area) were measured using an image analysis system WinSEEDLE™ Pro 2020a (Regent Instruments Inc., Sainte-Foy, Quebec, Canada). The data shown (Figure 1) represents the average of three replicates with each replicate measured twice using 100 grains. The 100-GW (g) was measured by random selection of 100 grains of each plant of each cultivar and carefully weighed on a digital scale with accuracy of 0.001 g.

### 4.3. Statistical Analysis and Phenotype Evaluation

The phenotypic data obtained for GL (mm), GW (mm), GLW ratio, % chalk and 100-GW were analyzed as a CRD following analysis of variance (ANOVA) using R statistical packages v4.1.0 [108] and John’s Macintosh Project (JMP) Genomics Pro version 12.0 for descriptive statistics. An ANOVA was carried out with a statistical model that included the effects of genotype, treatment (CDT and HDT stress) and the interaction between genotype and treatment. The Tukey’s Honest Significant Difference (HSD) test was used to compare the mean values of treatments among all the genotypes for significant effects (Tukey’s HSD, *p* < 0.05). Pearson’s correlation analysis between grain quality traits and 100-GW under both treatments (CDT and HDT stress) were performed using the package “corrplot” and were displayed in correlation plot using the package “ggplot2” [109] in R v4.1.0. The significance of the results was tested by the function cor.test [110] at the 95% confidence level.

### 4.4. Small RNA Libraries and Reads Analysis

The harvested spikelets containing R6 stage caryopsis were isolated from the 5 genotypes grown under CDT and HDT, flash frozen in liquid nitrogen, and stored at −80 °C until RNA was isolated. Three independent biological replicates for each genotype were collected from both CDT and HDT samples (a total of 30 samples) for sRNA-seq analysis. Total RNA was extracted using TRIzol reagent (Invitrogen, Carlsbad, CA, USA) according to the manufacturer’s protocol. The quality and quantity of total RNA were analyzed using Agilent Bioanalyzer 2100 (Santa Clara, CA, USA) and Nanodrop ND-1000 (Wilmington, DE, USA).

Small RNA Libraries were prepared according to TruSeq^®^ Small RNA Sample Preparation Guide (Illumina, San Diego, CA, USA) and sequenced on Illumina Novaseq™ 6000 platform. The sequenced reads quality was assessed using Fastqc [111] to confirm that adapters and low-quality reads had been successfully removed. Identical reads were pooled to generate non-redundant sequences (unique reads).

Rice genome sequences were downloaded from http://rice.uga.edu/downloads.shtml (accessed on 6 June 2022). Primary miRNA and mature miRNA sequences for rice were obtained from miRBase Release 22.1 [112]. Sequences for Osa-miR1867 and Osa-miR1867* were added manually due to the precise processing found in our samples and previous evidence that Osa-miR1867 targets starch synthase [23]. Noncoding RNA sequences for rice were extracted from Rfam 14.5 [113], NONCODE 6 [114] and GtRNAdb 7 [115].

The unique clean reads from each library were aligned to the rice genome, transcriptome, miRNA precursors and noncoding RNAs using bowtie [116]. In all cases, one mismatch was allowed for mapping. The option “–norc” was used when reads were mapped to the transcriptome, miRNA precursors or noncoding RNAs.

### 4.5. Identification of Novel microRNAs

To identify potential novel miRNA sequences from the sRNA libraries, ShortStack was used with the default settings to align the cleaned reads from each sRNA library to the rice genome [48]. For each sample, the ShortStack output GFF file was filtered to include only the clusters predicted to be miRNA precursors. Additionally, a GFF file for rice miRBase-annotated MIRNAs was downloaded. Bedtools intersect [117] was used to identify which predicted MIRNA loci had been previously recorded in miRBase. Predicted MIRNAs not overlapping with known MIRNA loci were considered as potential novel MIRNAs. To minimize false predictions, any potential miRNA sequence with less than 10 mature miRNA reads was discarded. For remaining sequences, the Mfold RNA folding server [118] was used to predict the hairpin structure. The hairpins were analyzed for compliance with the MIRNA criteria [119], which allows no more than five mismatches or three asymmetric bulges in the region of the predicted mature miRNA/miRNA* duplex. The processing precision was analyzed using a custom Python [120] script that calculates the ratio of reads that correspond to the mature miRNA, miRNA* and their 1nt positional variants at the 5p and 3p ends. For MIRNAs producing 20-22nt mature miRNAs, a ratio of at least 0.75 was required. For those producing 23-24nt mature miRNAs, a ratio of at least 0.90 was required, as high stringency is recommended for miRNAs of these lengths [119]. Finally, all potential novel miRNAs were required to pass all criteria in at least 4 libraries.

### 4.6. MicroRNA Quantification

The miRNA and miRNA* (miRNA-5p and miRNA-3p) sequences annotated at miRBase Release 22.1 were retrieved. For miRNAs where miRNA* is unavailable, miRNA* sequences were identified using the foldback structures so that the miRNA* complements the mature miRNA in the hairpin structure, with 2 nucleotide 3′ overhangs for both the miRNA and miRNA*. For ShortStack-discovered miRNAs, the 5p and 3p miRNA sequences were provided by the ShortStack output. Redundant miRNA/miRNA* sequences were combined.

Alignment of the small RNA libraries to the miRNA and miRNA* sequences was performed by aligning unique reads from sRNA libraries to the miRNA sequences using BLASTn [121]. For each unique sRNA read, the best BLAST alignment was kept if it had no more than 1 mismatch, no gaps and no more than a 1nt extension or truncation at 5′ or 3′. The counts for the unique sRNA reads were attributed to the matched miRNA where applicable, and the total counts for each miRNA were determined using an Rscript.

### 4.7. Validation of Differentially Expressed miRNAs

We used EdgeR to quantify miRNA expression changes under HDT for each genotype [122]. The miRNA counts were used as input, and the clean sRNA library sizes were used for normalization. Following EdgeR analysis, miRNAs with a *p*-value of under 0.05 were considered as differentially expressed miRNAs. The same criteria were used to detect differentially expressed miRNAs between Cypress and LaGrue grown under the same condition (CDT or HDT) and for the interaction term analysis.

The profiles of differentially expressed miRNAs were validated by using qRT-PCR of miRNA precursors. The precursor sequences were used to design primers. Total RNA was first reverse transcribed into cDNA using random primer and SuperScript™ II Reverse Transcriptase (Invitrogen, Carlsbad, CA, USA) according to the manufacturer’s instructions. The cDNAs were diluted 5 times, quantitative PCR was carried out using PowerUp™ SYBR™ Green Master Mix (Applied Biosystems™, Waltham, MA, USA) and LightCycler^®^ 96 Real-Time PCR System. Dissociation curves were checked to confirm specific amplifications. Three biological replicates and three technical replicates were used. U6 snRNA was used as reference to normalize the expression of MIRNAs. The same methodology was used to analyze the target genes of Osa-miR164, Osa-miR169 and Osa-miR444 with the exceptions that oligo-dT was used to prepare cDNA and that actin 2 was used to normalize the expression. MIRNA or mRNA expression under the CDT was set to 1.0, and the relative expression under HDT was determined using the comparative threshold cycle (2−ΔΔCT) method [123]. The sequence of the primers used in this study is presented in Table S14.

### 4.8. Data Visualization

Data visualization was performed in Rstudio [124]. Heatmaps were made using pheatmap [125], bar charts and volcano plots were generated with ggplot2 [109] and Venn diagrams were made using ggVennDiagram [126].

## 5. Conclusions

**Figure 6:**
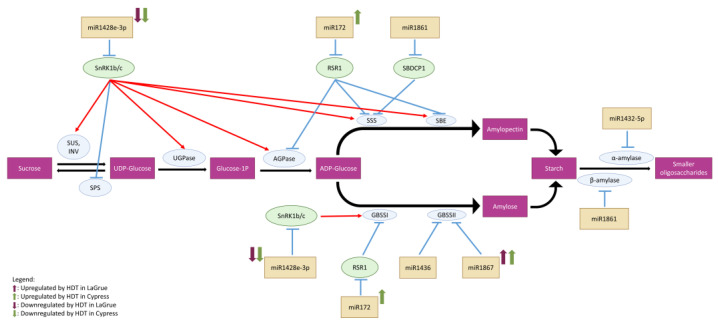
Simplified diagram of the starch metabolic pathway including miRNAs interacting with this pathway, and their regulation in R6 stage caryopsis samples under HDT in LaGrue and Cypress. Osa-miR1428e-3p, which is downregulated under HDT in both Cypress and LaGrue, inhibits starch accumulation by targeting *SnRK1b/c*. By phosphorylating, SnRK1b/c activates several starch biosynthesis enzymes such as sucrose synthase (SUS), invertase (INV), UDP-glucose pyrophosphorylase (UGPase), ADP-glucose pyrophosphorylase (AGPase), soluble starch synthase (SSS), glycogen bound starch synthase (GBSS) and starch branching enzyme (SBE). On the other hand, it inhibits the activity of sucrose phosphate synthase (SPS). Osa-miR172, which is upregulated under HDT in Cypress, promotes starch biosynthesis by degrading *RSR1*, a transcription factor that inhibits the expression of AGPase, SSS, GBSSI and SBE. Osa-miR1436 and Osa-miR1867, the latter of which is upregulated by HDT in Cypress and LaGrue, suppresses starch biosynthesis by inhibiting *GBSSII*. Osa-miR1861 promotes starch biosynthesis by inhibiting Starch Binding Domain-Containing Protein 1 (*SBDCP1*), which is a non-competitive inhibitor of SSIIIa. Osa-miR1861 and Osa-miR1432 also promotes starch accumulation by inhibiting beta-amylase and alpha amylase, respectively.

In conclusion, we compared miRNA profiles of the R6 stage caryopsis samples in five rice genotypes with contrasting chalkiness under HDT. As a whole, our analysis suggests that several miRNAs are abundantly expressed in R6 caryopsis tissue and are responding differently to heat stress. Most importantly, these regulations are largely genotype-dependent. This was particularly evident when comparing the miRNA regulation patterns of the low chalky Cypress with the genetically closely related, but high chalky, LaGrue. Making direct connection between miRNA regulation and HDT-induced chalkiness is difficult because the lack of substantial evidence for linking the known/predicted targets with the miRNAs in general. The very few direct links are through miR1436 and miR1867, both of which are predicted to target *GBSSII*, and miR1432 and miR1861, which could target starch degrading amylases (Figure 6, Table S15). However, HDT-regulated Osa-miR1428e-3p could more indirectly regulate starch metabolism by targeting *SnRK1b* and 1c. SnRK1 promotes starch biosynthesis by increasing the activities of several enzymes in this pathway [26]. HDT-responsive miRNAs likely decrease HDT-induced chalkiness through direct or indirect interaction with GRNs affecting starch and protein accumulation in the grain. This study suggests that Cypress may have better tendency to fine-tune miRNA expression levels that in turn could decrease chalkiness under HDT than the LaGrue. Further dissection of specific miRNA roles in this tissue could offer critical understanding as well as potential utility for engineering chalkiness under heat stress in rice genotypes.

## Figures and Tables

**Table 1:** Candidates for newly discovered miRNAs.

Name	Five Prime Sequence	Three Prime Sequence
canH ^1^-MIR11339	AGCAUUGCCCAUAUUCACAUAGAU	**AUAUAUGAAUGUGGCCAAUGCUAA** ^2^
canH-MIR169	AGCCAAGGAUGACUUGCCGGC	**CGGCAAGUUUGUCCUUGGCUAC**
canH-MIR2905	**GUCUUUAUCACUGACAUGUAGGCC**	CUCACAUGUCAAUGACAAAGGCAC
canH-MIR9774a	AGAAAUACCCAAUAUCUUGCUGA	**AGCAAGAUAUUGGGUAUUUCUUU**
canH-MIR9774b	AGAAAUACCCAAUAUCUUGCUGA	**AGCAAGAUAUUGGGUAUUUCUUU**
canN ^1^-01	**AUGUGGGUCGCUGACAUAUGGGCC**	CCCACAUGUCAGCCACUCAAAGUCA
canN-02	UACUCGAUUAGAUACCACCUAAUA	**CUAGGUGGUGUCUACUCGAGUUUU**
canN-03	**ACUCAGCCUAGGAGGGGAUGCGAC**	CACAUCCCCUCAUCGGUUGAGUUU
canN-04	GGACCAUCCACAUGUGAGGAGCUA	**AGUCCUCACGUGGGCAUAGUCUCC**
canN-05	UACUAGAGUUACUUCCACUUUGAA	**UAAAGUGUAGGCACCUCUAGUACA**
canN-06	**AAUGGCUUGUCUUGUUUUGUGUGC**	ACGCAAAACGAGCCAAGUCAUUAU
canN-07	**UCGCCGCGGCUGGCAUCAGCA**	CUGGCGCGAGCGGCGGCGAGG
canN-08	GAACUGCAUGGGAAAUUUUGUU	**CAAAAUUUUCCAUGCACUUCGA**
canN-09	**GAAGUGCAUGGGGAAUUUUGUU**	CGAAAUUUUCCAUGCACUUCGA

^1^ Newly identified miRNAs belonging to known families as labeled as “canH”, while novel miRNAs are labeled as “canN”. ^2^ The more abundant sequence (5p or 3p) is indicated in bold.

**Table 2:** Sequences of miRNAs analyzed in the quantitative RT-PCR analysis.

Name	Mature Sequence
Osa-miR164e	UGGAGAAGCAGGGCACGUGAG
Osa-miR169k	UAGCCAAGGAUGACUUGCCUG
Osa-miR169r	UAGCCAAGGAUGAUUUGCCUG
Osa-miR171e	UGAUUGAGCCGUGCCAAUAUC
Osa-miR444f	UGCAGUUGUUGCCUCAAGCUU
Osa-miR531ac	CUCGCCGGGGCUGCGUGCCGCCAU
Osa-miR1874	UAUGGAUGGAGGUGUAACCCGAUG
Osa-miR3979	CUUCGGGGGAGGAGAGAAGC

## Data Availability

The data presented in this study are openly available at the NCBI SRA database BioProject ID PRJNA970843.

## Supplementary Materials

The following supporting information can be downloaded at: https://www.mdpi.com/article/10.3390/ijms241411631/s1.
