# Supplementary material for: High Daytime Temperature Responsive MicroRNA Profiles in Developing Grains of Rice Varieties with Contrasting Chalkiness
Source: Int J Mol Sci. 2023 Jul 19;24(14):11631. doi: 10.3390/ijms241411631 (PMC10380806; doi:10.3390/ijms241411631)
Supplement: Supplementary file 1 [file ijms-24-11631-s001.zip › FigureS2.pdf]

canN-01

```
c--      g              G-|    C        A       gcga
   ucugac uacugcccucugAU  UGGGU GCUGACAU UGGGCCc    \
   ggauug augacgggagACUG  ACUCA CGACUGUA ACCCggg    g
cua      a              AA^    C        C       gauc
```

canN-01 Homologue in *Brachypodium distachyon*

```
----|   a  u                                 u  c   a
    guga gu acuucaccugacuuuggguggcugacauguggg cc acg \
    cacu ca ugaaguggacugaaacccaccgacuguacaccc gg ugc c
aagu^   g  u                                 -  u   c
```

canN-02

```
-  -|             a  uU      U             AUA    ga-       uu      ag  gua
 uc caaaauauagcaac ug  ACUCGA UAGAUACCACCUA   acua   gggucua  uagauu  ua   c
 ag guuuuauaucguug aU  UGAGCU AUCUGUGGUGGAU   ugau   cuuagau  aucuga  gu   u
u  c^             g  UU      C             Ca-    aug       cu      gg  gga
```

canN-03

```
---              ag            UAG         C   c   g    a-----|       a   a
   cucccuuuguccca  aaaaACUCAGCC   GAGGGGAUG GAC ccu cuag     gaaucugg cag g
   gagggaggcagggu  uuUUUGAGUUGG   CUCCCCUAC Cug gga gauc     cuuagacc guu g
uau              a-            CUA         A   a   g    auguug^       g   g
```

canN-04

```
a        c--  uuc         C  -| A          G  Aauc--   a        u    ugac
 ggcaggau   ac   uguggGGAC AU CC CAUGUGAGGA CU      cca ccaucuau uagc    \
 ucguccua   ug   auaCCUCUG UA GG GUGCACUCCU GA      ggu gguagaua auug    a
g        cuu  uau         A  C^ -          -  aucaau   c        c    ugua
```

canN-05

```
-        -| c    c    -        UUA   C       A  aa   ucag
 guuuuacg gu cuug aagu UACUAGAG   CUU CACUUUG Ag  aua    \
 caaaaugc ca gaac uuuA AUGAUCUC   GGA GUGAAAU uc  uau    c
a        a^ a    -    C        CAC   U       c  gc   uuac
```

canN-06

```
ua--    a  a        cc         U                 a     cac      --| g  cg
    uuca cu aucaugug  AAUGGCUUG CUUGUUUUGUGUGCcau aaaaa   uaggcu  ca uu  g
    aagu ga uaguacac  UUACUGAAC GAGCAAAACGCAcggua uuuuu   guucgg  gu ag  a
gaac    c  c        UA         C                 g     aa-      uu^ g  ua
```

canN-07

```
g---   g    ag     cc         G   G  AU    .-Auuca    u-   -   -|   c
    gcg cagc  gccau  ccUCGCCGC GCU GC  CAGC       gcag  ggc gca cggc g
    ugu gucg  cggua  GGAGCGGCG CGA CG  GUCg       cguc  ucg cgu gccg c
auga   g    g-     ca         G   G  CG    \ -----    uc   u   g^   c
```

canN-08

```
--|     cc  c     aua    C                U  gauu    a  gua-   c
  aucaag  au acaga   cGAA UGCAUGGGAAAUUUUG Ug    gguc gg    guc a
  uaguuu  ua ugucu   GCUU ACGUACCUUUUAAAAC gc    ccag cc    cag u
aa^     cu  c     aaA    C                c  agau    -  aagg   g
```

canN-09

```
--|     cc  c     aua            GG       U  gauu    a  gua-   c
  aucaaa  au acaga   cGAAGUGCAUGG  AAUUUUG Ug    gguc gg    guc a
  uaguuu  ua ugucu   GCUUCACGUACC  UUAAAGC ac    ccag cc    cag u
aa^     cu  c     aaA            UU       c  agau    -  aagg   g
```

canH-miR169

```
a---    c          a  c         UG -        .-C    g
    gagg agagagcagg ug AGCCAAGGA  A CUUGCCGG   cggc a
    cucu ucucucguuc aC UCGGUUCCU  U GAACGGCc   gccg u
ucug    -          c  A         GU U        \ -    g
```

canH-miR2905

```
-    c     -|  g            A   C        AG   c    u
 cucu uaguc acu caucguGUCUUU UCA UGACAUGU  GCC gaug \
 gaga gucag uga guggCACGGAAA AGU ACUGUACA  Cgg cugc g
g    c     c^  a            C   A        CU   -    u
```

canH-miR9774a

Initial ΔG = -37.80

```
           10        20        30        40
uu---| ug   a         cu                   U
     uu  aag aaggaaag  AGAAAUACCCAAUAUCUUGC G
     aa  uuu uuuuuuuU  UCUUUAUGGGUUAUAGAACG A
uuucu^ gu   a         U-                   A
     80        70         60        50
```

canH-miR9774b

Initial ΔG = -36.80

```
            10        20        30        40
uu----| ug   a         cu                   U
      uu  aag aaggaaag  AGAAAUACCCAAUAUCUUGC G
      aa  uuu uuuuuuUU  UCUUUAUGGGUUAUAGAACG A
cuuucu^ gu   a         --                   A
     80        70          60        50
```

canH-miR11339

Initial ΔG = -58.40

```
         10        20        30        40        50        60
--|   g              c        C         C   G     a           cacac
  uuca guuauaagauuuu uAGCAUUG CCAUAUUCA AUA AUguu augaaucuagg     a
  aagu uaauauucugaaa AUCGUAAC GGUGUAAGU UAU uacaa uacuuagaucu     u
ca^   a              A        C         A   A     c           auaua
130       120       110       100         90        80        70
```
