# Supplementary material for: High Daytime Temperature Responsive MicroRNA Profiles in Developing Grains of Rice Varieties with Contrasting Chalkiness
Source: Int J Mol Sci. 2023 Jul 19;24(14):11631. doi: 10.3390/ijms241411631 (PMC10380806; doi:10.3390/ijms241411631)
Supplement: Supplementary file 1 [file ijms-24-11631-s001.zip › FigureS3.pdf]

```
canH-miR169          1  agccaaggaugacuugccgg  20
                        ||||||||||||||||||||
osa-miR169b/c/e      2  agccaaggaugacuugccgg  21


canH-miR11339-5p     2  gcauugcccauauucacauag  22
                        |||||||||| ||||| ||||
osa-miR11339-5p      1  gcauugcccacauucauauag  21


canH-miR2905-3p      4  acaugucaaugacaaaggca  23
                        |||||||| |||||||||||
osa-miR2905          2  acaugucagugacaaaggca  21


canH-miR9774ab-3p    3  caagauauuggguauuucuuu  23
                        ||||||||||||||||||| |
tae-miR9774          1  caagauauuggguauuucugu  21
```
