# Supplementary figures and images for: High Daytime Temperature Responsive MicroRNA Profiles in Developing Grains of Rice Varieties with Contrasting Chalkiness
Source: Int J Mol Sci. 2023 Jul 19;24(14):11631. doi: 10.3390/ijms241411631 (PMC10380806; doi:10.3390/ijms241411631)

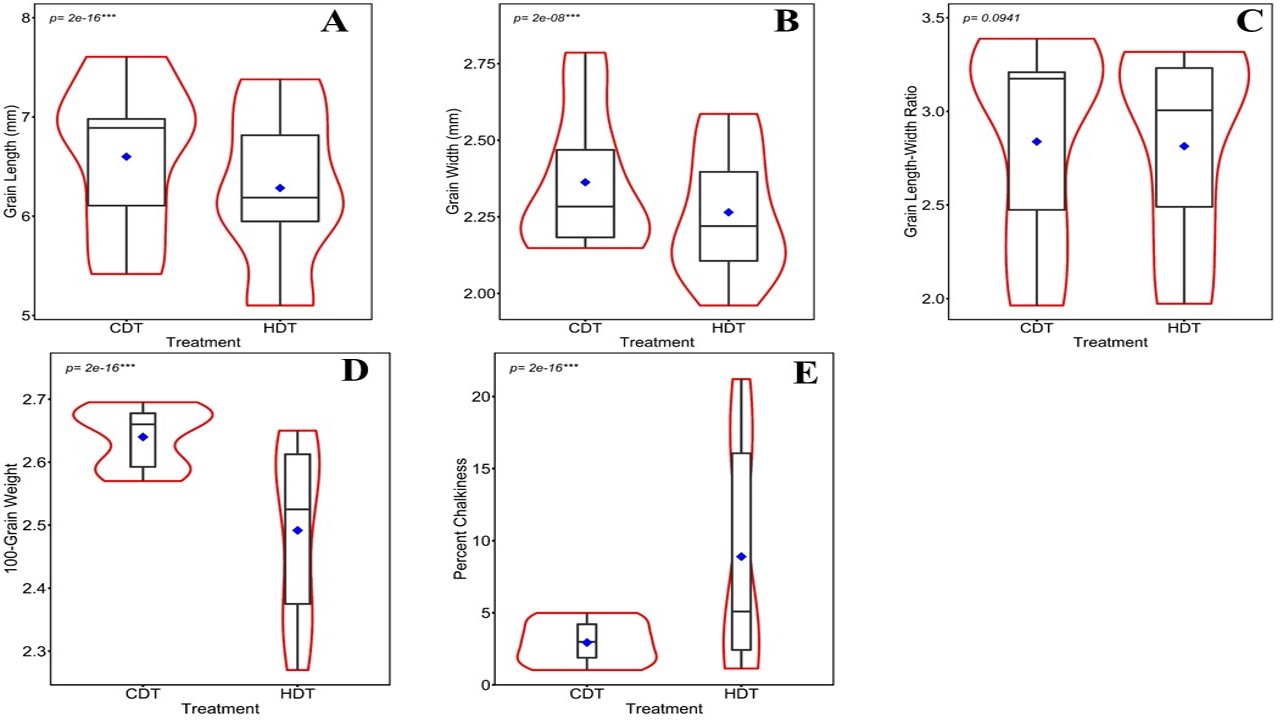

Supplement: Supplementary file 1 [file ijms-24-11631-s001.zip › FigureS1.JPG]

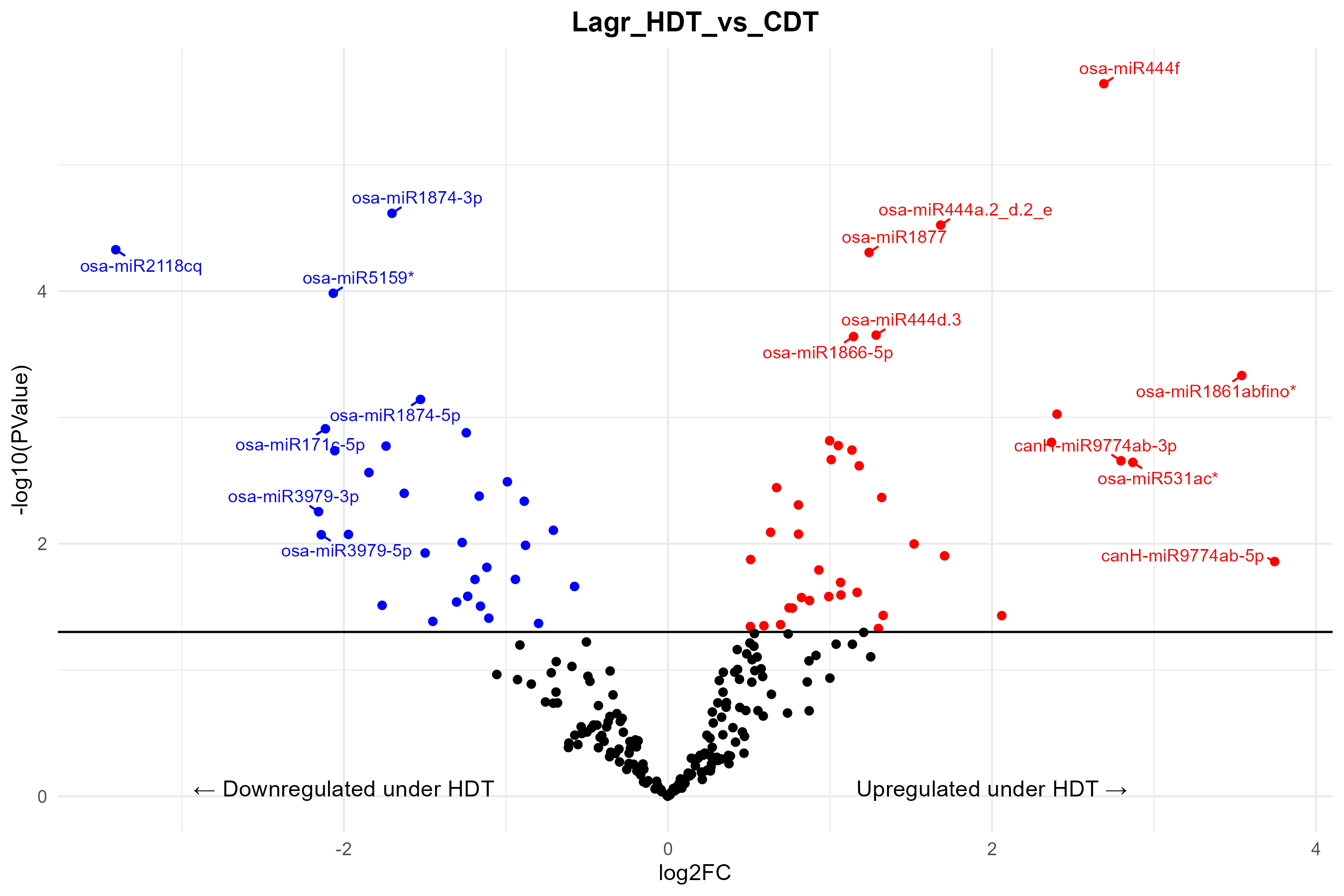

Supplement: Supplementary file 1 [file ijms-24-11631-s001.zip › FigureS4.tiff]

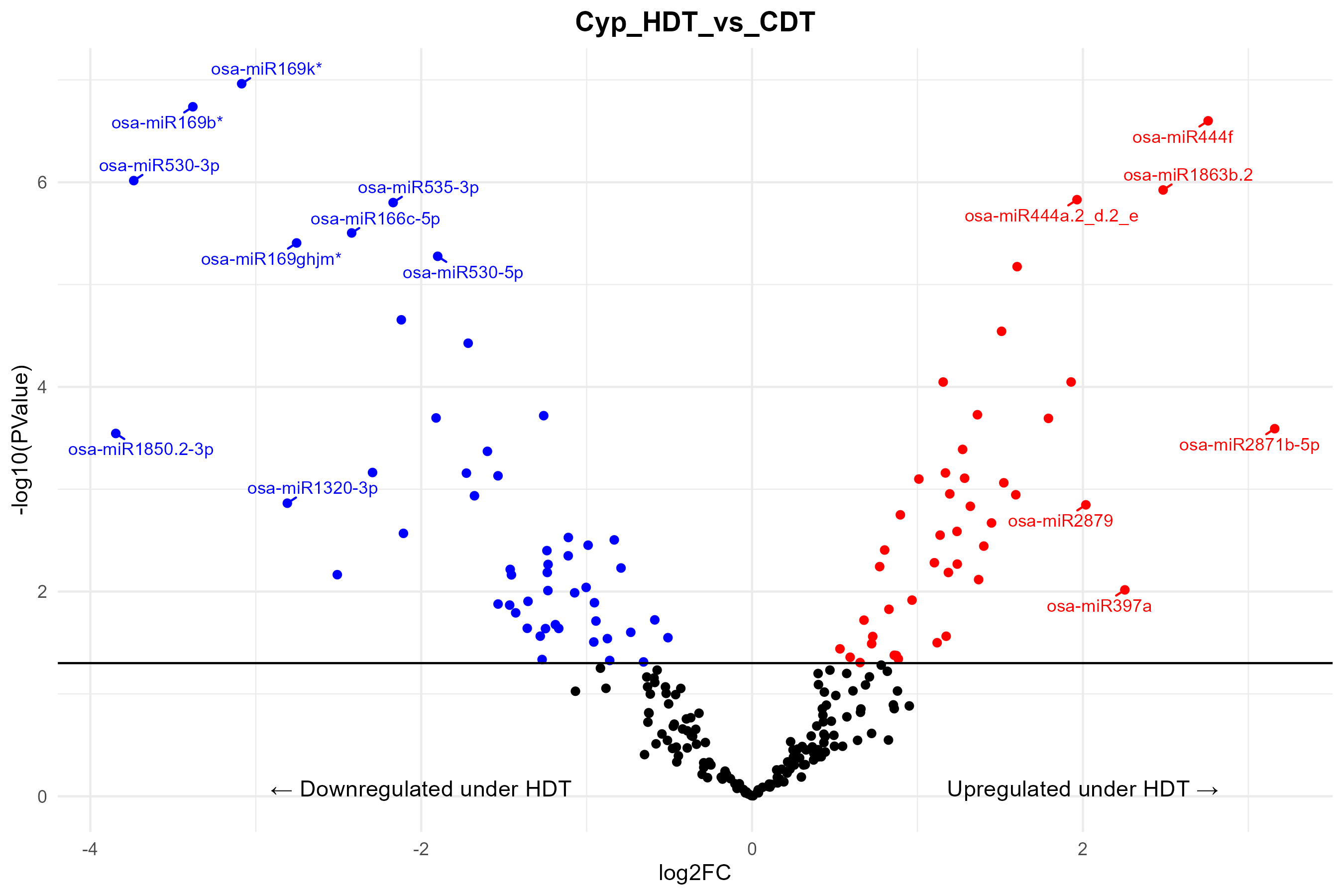

Supplement: Supplementary file 1 [file ijms-24-11631-s001.zip › FigureS5.tiff]

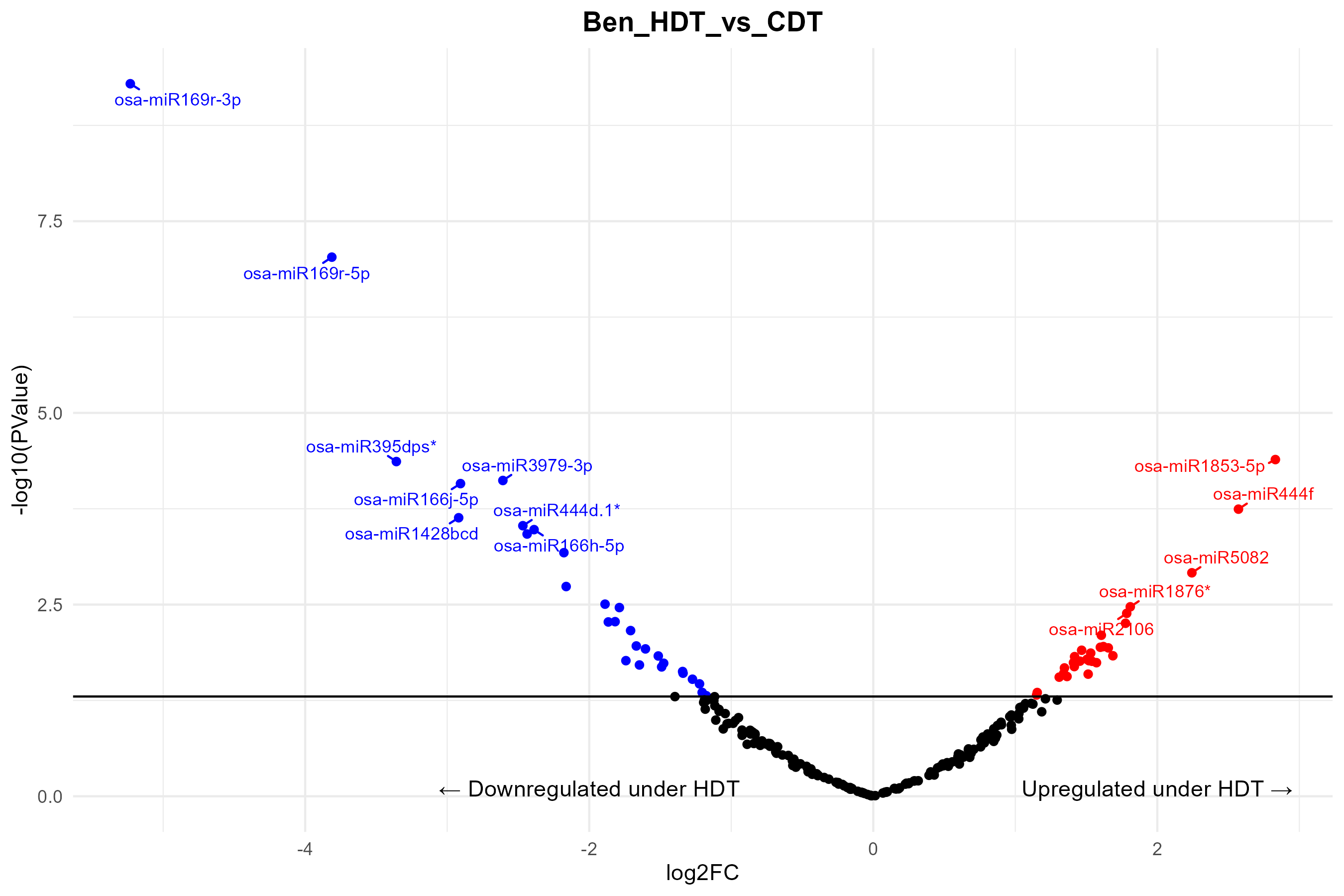

Supplement: Supplementary file 1 [file ijms-24-11631-s001.zip › FigureS6.tiff]

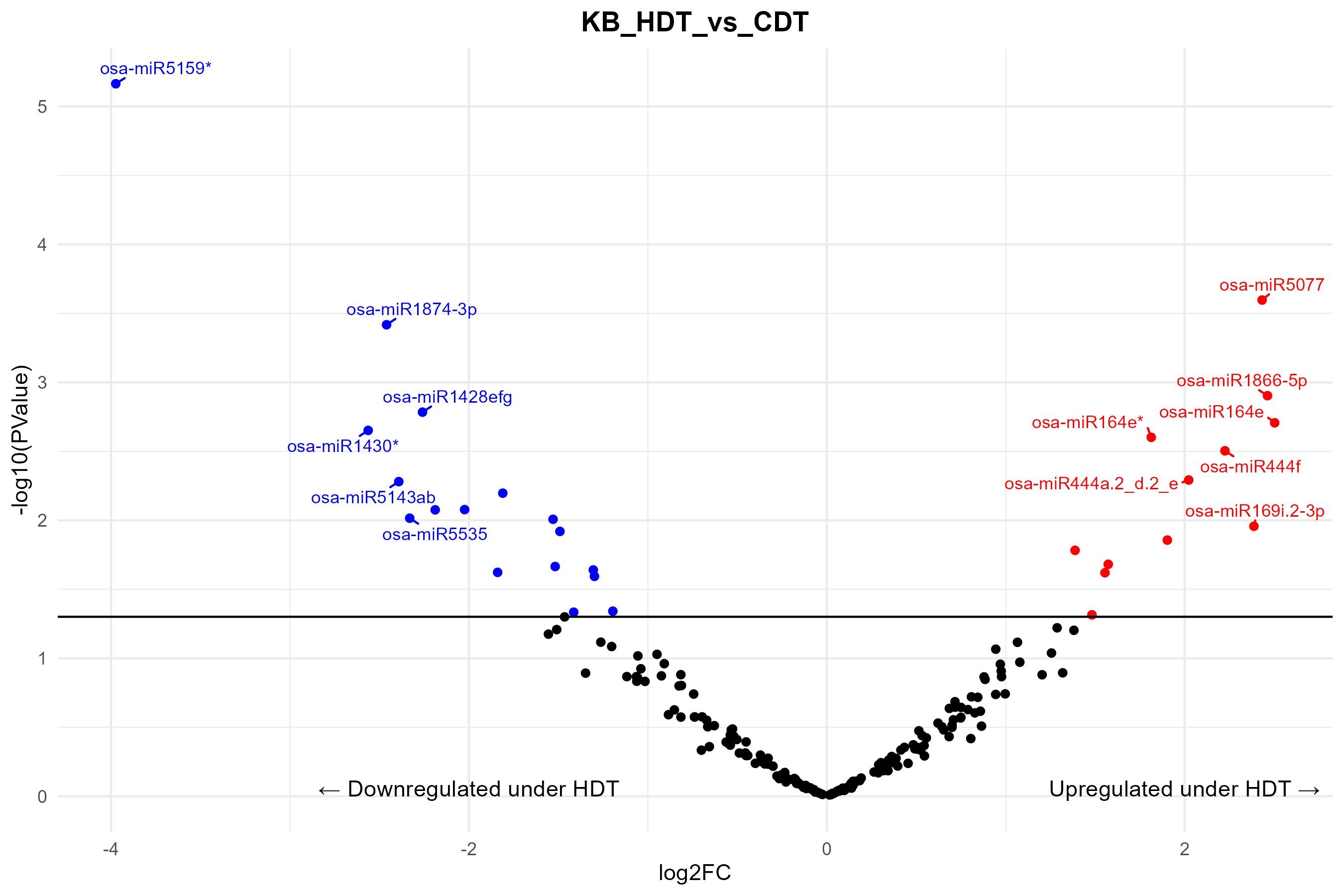

Supplement: Supplementary file 1 [file ijms-24-11631-s001.zip › FigureS7.tiff]

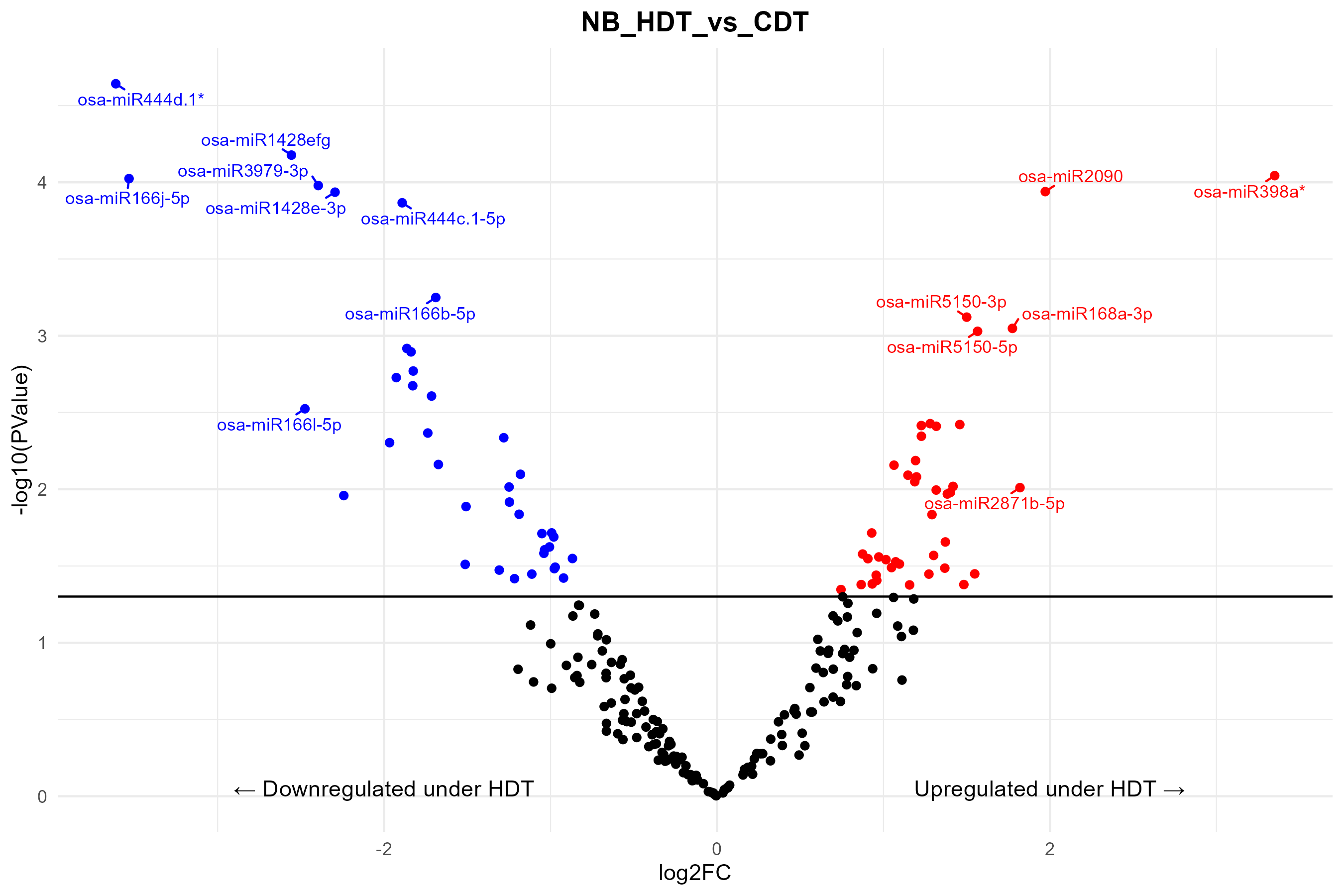

Supplement: Supplementary file 1 [file ijms-24-11631-s001.zip › FigureS8.tiff]

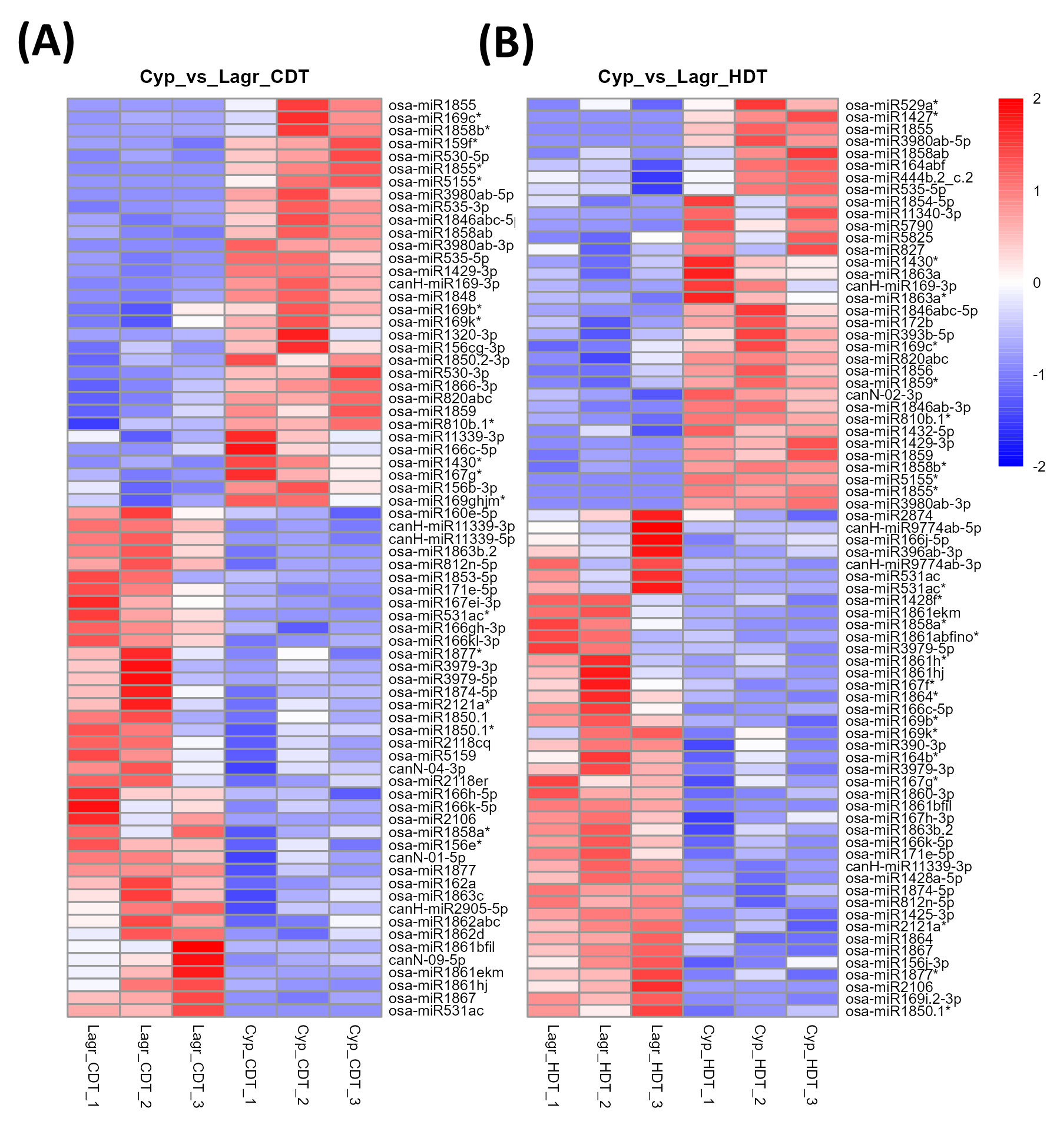

Supplement: Supplementary file 1 [file ijms-24-11631-s001.zip › FigureS9.tiff]
